# Electrical and Myocardial Remodeling in Primary Aldosteronism

**DOI:** 10.3389/fcvm.2014.00007

**Published:** 2014-11-06

**Authors:** Mario Curione, Luigi Petramala, Claudio Savoriti, Marisa Verrenti, Erika Baiocco, Stephanie Salvatore, Laura Zinnamosca, Gino Iannucci, Susanna Sciomer, Claudio Letizia

**Affiliations:** ^1^Department of Internal Medicine and Medical Specialties, Sapienza University of Rome, Rome, Italy; ^2^Specialized Center of Secondary Hypertension, Sapienza University of Rome, Rome, Italy

**Keywords:** hyperaldosteronism, essential hypertension, electrocardiography, left ventricular hypertrophy, conduction disturbance

## Abstract

**Objective and design:** Primary aldosteronism (PA) represents the most common cause of secondary hypertension. A higher risk of cardiovascular events has been reported in patients with PA than in otherwise similar patients with essential hypertension (EH). So far, only a few studies investigated the electrocardiographic changes in PA patients compared to EH patients.

**Methods:** To investigate the electrocardiographic changes and heart remodeling in PA, we enrolled 61 consecutive patients, 30 with PA [12 with aldosterone-producing adrenal cortical adenoma (APA) and 18 with bilateral adrenal hyperplasia-idiopathic adrenal hyperplasia] and 30 with EH. In all subjects, electrocardiographic parameters were evaluated from 12-lead electrocardiograms and heart remodeling with echocardiogram.

**Results:** No significant differences in age, sex, body mass index, and blood pressure were found in two groups. The *P* wave and PR interval duration were significantly prolonged in patients with PA respect to EH (*p* < 0.003 and <0.002, respectively). A first degree atrio-ventricular block was present in 16% of the patients with PA and only in 3.2% of those with EH. In PA patients, the interventricular septum thickness (IVST) correlated with PR duration (*r* = 0.51; *p* < 0.03). Left ventricular hypertrophy was present in 53% of the patients with PA and in 26% of the patients with EH (χ^2^, *p* < 0.03).

**Conclusion**: In this case–control study, patients with PA show more anatomic and electrical heart remodeling than those with EH. We hypothesize that in patients with PA these cardiac changes may play a role for the increased risk of future cardiovascular events.

## Introduction

Primary aldosteronism (PA) is a common cause of secondary arterial hypertension accounting for 5–15% of all hypertensive patients in special centers (1–5). It is of particular importance to diagnose or rule out this condition, as hypertensive patients affected by PA can be cured with adrenalectomy or appropriate medical therapy (mineral-corticoid receptor (MR) antagonists) in case of APA and/or idiopathic adrenal hyperplasia (IHA) (6). This is relevant, as it has been demonstrated that patients with aldosterone excess are more prone to stroke, myocardial infarction, and atrial fibrillation than are essential hypertensives (EH) with risk profiles (7, 8).

Several studies have shown that patients with PA have a higher risk for target organ damage than other EH patients [such as left ventricular hypertrophy (LVH), QT interval prolongation, and prevalence of metabolic syndrome (SM)] (9–11). This effect is partially independent of blood pressure change (12), but probably determined by the effect of aldosterone excess (13).

These non-epithelial effects of aldosterone supports the concept that blood pressure and hemodynamic overload are not only factors involved in the genesis of organ damage such as LVH. In fact, aldosterone contributes to cardiovascular remodeling via non-genomic processes, locally synthesized in blood vessel contributes to the hypertrophy of vascular smooth cells (VMMC) (14), and determined alteration to the geometry of myocardial cells with cell proliferation and fibrosis (15).

Moreover, many studies have been performed to evaluate the heart remodeling in PA patients, but until now only few studies have investigated the electrocardiographic changes in those patients independent at the levels of serum potassium.

Based on the electrophysiological aldosterone modifications in cardiac myocytes and fibers of conduction system (16), the aim of this study was to investigate electrocardiographic changes and heart remodeling in PA patients compared to EH patients.

## Materials and Methods

### Patients

Sixty-one adult patients (39 males and 22 females) were enrolled: 30 patients with PA (12 with APA and 18 with IHA) and 31 patients with EH. All patients have been referred to the Specialized Center of Secondary Hypertension, Department of Internal Medicine and Medical Specialities, University of Rome “Sapienza”, Italy.

This study was approved by our ethical committee, and informed consent was obtained from all subjects before their enrollment.

### Diagnosis of primary aldosteronism

After 2 weeks of following a normal sodium (140–150 mEq/day) and potassium intake (40–50 mEq/day), fasting blood samples for plasma aldosterone concentration (PAC) and plasma renin activity (PRA) were obtained in all subjects.

Patients were supine for at least half an hour. All anti-hypertensive drugs were withdrawn at least 3 weeks (up to 2 months for anti-aldosterone drugs) before hematochimic, biochemical, and hormonal evaluation.

In hypertensive patients whose treatment could not be withdrawn for ethical reasons, calcium-channel blockers (nifedipine or amlodipine) or α-1-receptor blockers (doxazosina) were utilized at doses required to achieve blood pressure control. These agents are considered to have a neutral effect of rennin–angiotensin aldosterone system (RAAS).

Diagnosis of PA was made as described previously (10). A cut-off PAC/PRA ratio of more than 40 (ng/dl: ng/ml/h) in the presence of PAC higher than 15 ng/dl and suppressed PRA (≤0.2 ng/ml/h) was used as a screening for PA. In the case of a PAC/PRA ratio greater than 40 (ng/dl:ng/ml/h), patients underwent a saline infusion (0.9% NaCl 500 ml/h for 4 h) as a confirmatory test, and only those with PAC levels that failed to decrease to <5 ng/dl after the saline infusion were diagnosed as having PA. An imaging test comprising high-resolution computed tomography (CT) with 3 mm slices and systematic use of a contrast medium and/or magnetic resonance imaging (MRI) was mandatory in all patients with a positive test and PA ≥5 ng/dl after saline infusion. In all cases, the diagnosis of APA was confirmed by histopathological diagnosis, while IHA diagnosis was performed by technical imaging (e.g., adrenal vein sampling).

### Essential arterial hypertension

The diagnosis of EH was established based on the absence of clinical history and laboratory data of secondary hypertension: principally normal values or plasma and urinary electrolytes, renal function, urinary metanephrines, the RAAS, urinary free cortisol, and calcium-phosphorus system. All patients with white coat hypertension were excluded from the study.

### Electrocardiogram

All patients underwent a 12-lead resting electrocardiogram (ECG) with PC ECG NORAV 1200 MEDICAL Ltd. using a specific software, the following parameters have been calculated: amplitude and duration of *P* wave, PR interval duration, and amplitude and duration of QRS complex, QT interval, QT interval corrected for the previous cardiac cycle length (QTc) by Bazett formula and QT dispersion (QTd).

### Echocardiogram

All patients underwent transthoracic echocardiograms performed by the same operator with 3.5 MHz probe, II armonica, Toshiba Sonovue 8000. Two different readers measured all of the echocardiograms, in a blind manner, the average of two calculations was considered for the study of the left ventricular (LV) internal dimensions, interventricular septum (IVS), and posterior wall thickness were measured according to the recommendations of the American Society of Echocardiography (17) and derived anatomic variables were calculated (15). Left ventricular mass index (LVMi) was obtained by normalization of LVM for height to the 2.7 power, and LVH was prospectively defined as a value of LVMi ≥50 g/m^2.7^ in males and 47 g/m^2.7^ in females (18). Ejection fraction, endocardial, and midwall fractional shortening (FS) were calculated by standard methods (19). LV end-diastolic and end-systolic values were calculated with the Teicholz’s correction of the cube formula (20).

### Diagnosis of metabolic syndrome

Metabolic syndrome was defined according to Adult Treatment Panel III (21) criteria, and its diagnosis required three or more of the following conditions: (1) waist circumference >102 cm in men and >88 cm in women, (2) triglycerides of 150 mg/dl or higher, (3) high-density lipoprotein (HDL)-cholesterol <40 mg/dl for men and <50 mg/dl for women, (4) fasting glucose of 100 mg/dl or more, and (5) systolic BP of 130 mmHg or more and diastolic BP of 85 mmHg or more.

### Biochemical measurements

Plasma renin activity was measured by radioimmunoassay (RIA) using commercial kits (RenCTK: Sorin Biometrics). Normal range sitting at rest, on normal sodium diet was 0.2–2.8 ng/ml/h; intra-assay and inter-assay coefficients of variations (CVs) were within 8 and 10%, respectively. The assay for PAC was performed with diagnostic kits (Aldosterone Mirya, Technogenetics). Normal range was 1–15 ng/dl supine, 3–32 ng/dl upright on a normal sodium diet; intra-assay and inter-assay CV, where both <5–6%; the cross-reactivity of the antibody for aldosterone for other adrenal steroids was <0.001%.

### Exclusion criteria

Patients with non-sinus rhythm, advanced atrio-ventricular block, bundle branch block, coronary artery disease (CAD), structural abnormalities of heart and those on antiarrhythmic, ACE-inhibitor or angiotensin receptor blocker therapy, renal failure (GFR-MDRD <60 ml/min), liver failure, diabetes, peripheral artery disease (ABI < 0.9), hypo-hypercalcemia, hypo-hypermagnesiemia were excluded.

### Statistical analysis

Saphiro Wilk test was used to check the assumption of normality of the variables. Student’s test was used for the two groups’ comparison of continuous variables, while χ^2^ test was used for categorical variables. Person’s rho was calculated to analyze association between electrocardiographic parameters; the dependent variables have been the following factors: sex, age, body mass index (BMI), SM, smoke, serum potassium levels, as independent variables. All statistical comparisons were performed using two-tailed significant tests, with *p* ≤ 0.05, was considered statistically significant. STATA was used for all analysis.

## Results

Demographic and hemodynamic data are reported in Table 1. No significant differences for age, gender, BMI, systolic and diastolic blood pressure, and heart rate were found between PA and EH patients. Moreover, we did not reveal a significant prevalence in both groups for SM (Table 1).

**Table 1:** **Statistical differences in demographic characteristics and laboratory data in two groups of patients (values are expressed as mean ± SD)**.

Parameter	PA (*n* = 30)	EH (*n* = 31)	*p*
Age (years)	55 ± 11	54 ± 10	NS
Gender (male/female)	8/22	16/14	NS
Body mass index (kg/m^2^)	26.9 ± 4	25.1 ± 3	NS
SBP/DBP	135 ± 17/82 ± 10	129 ± 11/79 ± 6	NS/NS
Heart rate (bpm)	70 ± 10	70 ± 8	NS
Glycemia (mg/dL)	92 ± 13	94 ± 18	NS
Metabolic syndrome	11/30	8/31	NS
Serum K^+^ (mEq/dL)	3.99 ± 0.68	4.27 ± 0.37	<0.05
Creatinine (mg/dL)	1 ± 0.19	0.86 ± 0.15	<0.001
PAC (ng/dl)	426 ± 422	104 ± 60	<0.0001
PAC/PRA (ng/dl:ng/ml/h)	84.3 ± 79	17.6 ± 15	<0.00002

*PA, primary aldosteronism; EH, essential hypertension; SBP/DBP, systolic and diastolic blood pressure; PAC, plasma aldosterone concentration; PAC/PRA, plasma aldosterone/plasma renin activity; NS, not significant; *p* < 0.05 is considered statistically significant*.

As shown in Table 1, serum potassium levels were significantly different between PA patients (3.99 ± 0.68 mEq/l) and EH patients (4.27 ± 0.37 mEq/l) (*p* < 0.05, respectively). Moreover, in patients with PA, we found a significant increase of serum creatinine values (1 ± 0.19 mg/dl) compared to EH patients (0.86 ± 0.15 mg/dl) (*p* < 0.001, respectively). As expected, PAC and PRA levels were significantly altered in PA patients compared to EH patients, and in particular PAC/PRA ratio was significantly increased (84.3 ± 79 vs. 17.6 ± 15 ng/dl:ng/ml/h; *p* < 0.0002, respectively).

The *P* wave and PR interval duration were significantly prolonged in patients with PA compared to EH patients (*p* < 0.003 and <0.02, respectively) (Table 2). In Table 3, we reported the statistical differences of the echocardiographic parameters in the two study groups.

**Table 2:** **Statistical differences in electrocardiographic parameters in two groups of patients (values are expressed as mean ± SD)**.

Parameters	PA (*n* = 30)	EH (*n* = 31)	*p*
*P* wave (ms)	108.7 ± 13.7	92.4 ± 12.8	<0.01
PR interval (ms)	177.9 ± 30.6	152.3 ± 19.5	<0.02
QRS interval (ms)	85.5 ± 21.4	75.2 ± 11.5	<0.04
QTc interval	406.7 ± 29.8	404.9 ± 16.0	NS
QTd	41.4 ± 29.0	38.9 ± 22.5	NS

*PA, primary aldosteronism; EH, essential hypertension; *p* < 0.05 is considered statistically significant*.

**Table 3:** **Statistical differences of the echocardiographic parameters in two groups of patients (values are expressed as mean ± SD)**.

Parameters	PA (*n* = 30)	EH (*n* = 31)	*p*
LVM (g)	218.7 ± 53.5	185.9 ± 42.3	<0.01
LVMi (g/m^2.7^)	50.7 ± 11.4	45.2 ± 10.1	<0.05
IVS (mm)	11.3 ± 1.3	10.0 ± 1.2	<0.01
PW (mm)	10.7 ± 1.4	9.6 ± 1.2	<0.01

*PA, primary aldosteronism; EH, essential hypertension; LVM, left ventricular mass; LVMi, left ventricular mass index; IVS, interventricular septum thickness; PW, posterior wall thickness; *p* < 0.05 is considered statistically significant*.

**Figure 1:**
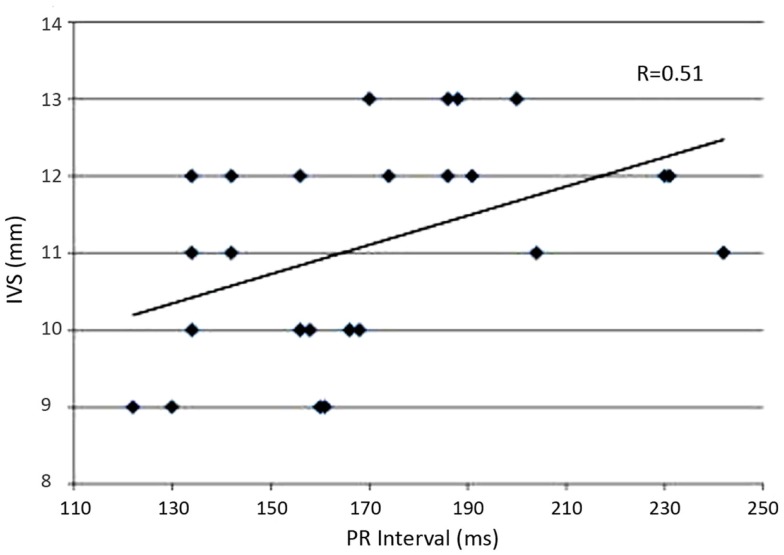
**The graph represents the linear regression between interventricular septum (IVS) wall thickness and PR interval duration in patient with primary aldosteronism**.

First degree atrio-ventricular block was present in 16% of PA patients, but only in 2–3% patients with EH (*p* = 0.09). In PA patients, PR duration positively correlated with IVS (*r* = 0.51, *p* < 0.03), and IVS with LVMi (*r* = 0.54, *p* < 0.04) (Figure 1).

Primary aldosteronism patients showed a significant increase of the QRS duration compared to EH patients (*p* < 0.04). This increase was not significantly revealed in PA patients when multivariate analysis was performed.

QTc interval, calculated according to Bazett’s Formula, did not differ significantly between the two groups. However, patients with corrected QTc >440 ms were affected by PA. Finally, the degree of QTd in PA patients remained unchanged compared with EH patients (Table 2).

## Discussion

In the present study, we demonstrated a positive correlation between PR interval and both IVT and LVMI cardio-parameters in patients with PA, and we hypotheses that the electrical abnormalities can be related to myocardial remodeling in this disease secondary, in part, to aldosterone hypersecretion.

In the last years, many studies have been performed in PA in order to investigate the effects of aldosterone on cardiovascular system, in terms of CAD, LV function, arterial stiffness, and arrhythmias (7, 8, 12).

For a long time, PA has been considered a relatively benign form of arterial hypertension, but recent studies suggest that chronic exposure to aldosterone might lead to cardiovascular and renal damage (11). In fact, PA patients display unfavorable cardiovascular profiles, suggesting that aldosterone may play an additional role beyond its well-known hypertensive effects. Extensive experiments using animal models have demonstrated that aldosterone co-stimulate an abnormal accumulation of collagen (type I and Type II), which can be reversed partially by a treatment with spironolactone, an MR antagonists. In particular, a study by Brilla et al. (17) demonstrated that an aldosterone infusion with a high salt-intake induces both cardiac hypertrophy and myocardial interstitial fibrosis. In clinical studies, Rossi et al. (15) reported that PA patients exhibit significant changes in the myocardial tissue as compared to EH patients, and secondary to cardiac fibrosis, can serve as an important determinant of myocardial remodelling and impaired tissue stiffness. Thus, patients with PA exprimed more cardiovascular alterations that EH patients, not only due to blood pressure, suggesting that aldosteronism status represents “*per se*” an independent factor for cardiovascular modifications with deposition of collagen fibers in myocardial texture and electrical cardiac conduction (7). Three mechanisms have been proposed to explain the slow cardiac conduction secondary, in part, to myocardial fibrosis: (1) reducing sodium current; (2) reducing cell-to-cell coupling with down-regulation of the number of connexins (such as CX43), and (3) barriers of micro-fibrosis that reduces transfer coupling (16).

Our previous study showed that PR interval was particularly large in PA patients compared to EH patients. In particular, PR interval prolongation was positively correlated with IVS thickness, but PR interval could be also related to hypokalemia or specific pharmacological therapy. Potassium plays an important role in maintaining the electrical potential across the cellular membrane, as well as in the depolarization and repolarization of the myocytes. In particular, hypokalemia can have dramatic effects on the cardiac cell conduction and may lead to observable changes on the ECG. Moreover, as the serum potassium levels delaine, the transmembrane is an elevation in the resting membrane potential and a prolongation of the action potential (particularly in phase 3 repolarization) and the refractory periods (18). As a result of the increased duration of the activation potential and the refractory period, patients with hypokalemia have an increased risk for arrhythmias.

Regarding the drugs treatment for arterial hypertension, in our study all patients (PA and EH) were treated with dihydropiridine calcium-antagonists and/or α-1 blockers which do not have particular effects on cardiac conduction system. Moreover, in our study, an increase of QTc length (even into range normality) was found in PA patients compared to EH patients. Additionally, we found a positive correlation between QTc prolongation and PAC levels in PA patients. The data were, in part, similar with the one reported by Maula et al. (9), which revealed that almost half of the patients with PA exhibited a pathological QTc length (>440 ms).

We speculated that aldosterone effect through non-genomic pathway, associated with an increase of second biochemical messengers, such as calcium or inositol triphosphate, may determine a prolongation of cardiac potential. Cardiac ion channels have lipophilic domain capable to bind steroid hormone with consequently activation or blocking ion channels themselves. Ion causes alteration, though a prolongation of 2 and/or 3 phase of potential action can increase the generation of post-repolarization potentials and a QT prolongation, with potentially ventricular arrhythmias (19). A difference in QTd was non-found in our study groups, which means that a homogenous repolarization was preserved that these data were in argument with another study performed by Youg et al. (20) that revealed no significant difference for QTd prolongation in PA and EH groups. Moreover, it demonstrated that pharmacological treatment with spironolactone decreases QTd in patients with heart failure, and reduces the risk of sudden death in patients with infarction myocardial acute (IMA) and heart failure (21–23).

Finally, we revealed a significant increase of LVHi in our patients with PA compared to EH patients. These results confirm and extend other studies reported in the literature (24–27). The LVH to the increased after-load of patients with high blood pressure by developing LVH, which predicts cardiovascular events (28) and should be regressed to improve prognosis (29). Aldosterone is the key to LVH development: apart from affecting pre-load and after-load in the setting of a high sodium intake, it augments the effects of angiotensin II on AT-1 receptors (30, 31), and also causes inflammation and fibrosis (30). Altogether, these actions can explain both the worse prognosis of patients with PA compared to EH patients (7) and the survival benefit conformed by MR antagonists in patients with LV remodeling (21, 22).

## Author Contributions

Conception and design of the work: Mario Curione, Claudio Letizia. Acquisition of data: Luigi Petramala, Claudio Savoriti, Marisa Verrenti, Erika Baiocco, Stephanie Salvatore, Laura Zinnamosca, Gino Iannucci, Susanna Sciomer. Analysis of data: Claudio Savoriti, Marisa Verrenti, Erika Baiocco, Stephanie Salvatore, Laura Zinnamosca, Gino Iannucci, Susanna Sciomer. Interpretation of data: Mario Curione, Luigi Petramala, Claudio Letizia. Draft of the work: Luigi Petramala, Claudio Savoriti, Marisa Verrenti, Erika Baiocco, Stephanie Salvatore, Laura Zinnamosca, Gino Iannucci, Susanna Sciomer. Revision of the work: Mario Curione, Claudio Letizia. Final approval: Mario Curione, Luigi Petramala, Claudio Savoriti, Marisa Verrenti, Erika Baiocco, Stephanie Salvatore, Laura Zinnamosca, Gino Iannucci, Susanna Sciomer, Claudio Letizia. All Authors agree the accuracy and integrity of any part of the work.

## Conflict of Interest Statement

The authors declare that the research was conducted in the absence of any commercial or financial relationships that could be construed as a potential conflict of interest.
